# *In vitro* Anti-Hantavirus Activity of Protein Kinase Inhibitor 8G1 Targeting AKT/mTOR/eIF4E Signaling Pathway

**DOI:** 10.3389/fmicb.2022.880258

**Published:** 2022-06-30

**Authors:** Zhoupeng Li, Fang Wang, Qikang Ying, Dehui Kong, Xiaoxiao Zhang, Yuhang Dong, Yongsheng Liu, Dongsheng Zhai, Zhou Chen, Min Jia, Xiaoyan Xue, Mingkai Li, Xingan Wu

**Affiliations:** ^1^Department of Pharmacology, School of Pharmacy, The Fourth Military Medical University, Xi'an, China; ^2^Department of Microbiology, School of Basic Medicine, The Fourth Military Medical University, Xi'an, China; ^3^School of Nursing, Army Medical University, Third Military Medical University, Chongqing, China; ^4^Precision Pharmacy and Drug Development Center, The Fourth Military Medical University, Xi'an, China

**Keywords:** Hantaan virus, protein kinase, inhibitor, the mammalian target of rapamycin, nucleocapsid protein

## Abstract

Hantaan virus (HTNV) is the main cause of hemorrhagic fever with renal syndrome (HFRS) around the world, which results in profound morbidity and mortality. However, there are currently no FDA-approved therapeutics or vaccines against HFRS. To find new anti-HTNV drugs, the inhibitory activity of 901 small molecule kinase inhibitors against HTNV is analyzed. Among these compounds, compound 8G1 inhibits HTNV with a relatively high inhibition rate and lower toxicity. The viral titer and nucleocapsid protein of HTNV are reduced after compound 8G1 treatment in a dose-dependent manner at concentrations ranging from 1 to 20 μM. In addition, the administration of compound 8G1 at the early stage of HTNV infection can inhibit the replication of HTNV. The molecular docking result reveals that compound 8G1 forms interactions with the key amino acid residues of serine/threonine-protein kinase B (Akt), which is responsible for the observed affinity. Then, the mammalian target of rapamycin (mTOR) and eukaryotic translation initiation factor 4E (eIF4E) signaling pathways are inhibited. Our results may help to design novel targets for therapeutic intervention against HTNV infection and to understand the anti-HTNV mechanism of protein kinase inhibitors.

## Introduction

Hantaviruses, which belong to the order *Bunyavirales*, are globally emerging pathogens transmitted by rodents to humans (Serris et al., 2020). Hantaviruses have a worldwide distribution and are categorized into two groups based on geography and pathobiology, namely, New World hantaviruses (NWH), such as Andes virus (ANDV) and Sin Nombre virus (SNV), which cause hantavirus cardiopulmonary syndrome (HCPS); and Old World hantaviruses (OWH), such as Seoul virus (SEOV) and Hantaan virus (HTNV), which cause hemorrhagic fever with renal syndrome (HFRS; Engdahl et al., 2021).

Hantaviruses are enveloped, negative-sense, single-stranded RNA viruses with a tripartite genome coding nucleocapsid protein (NP), glycoprotein precursor (GPC), and viral RNA-dependent RNA polymerase (RdRp; Wang et al., 2019). Infection induced by this virus in humans can result in two clinical syndromes: HFRS and HCPS. It was reported that over 50,000 cases occur globally each year, with fatality rates of up to 12% (HFRS) and 40% (HCPS; Watson et al., 2014). In recent years, there has been an improved understanding of the epidemiology following an increase in the number of outbreaks in China. HFRS was mainly caused by the HTNV (Zheng et al., 2018; Lu et al., 2020), which is responsible for thousands of HFRS cases annually (Avsic-Zupanc et al., 2019). In part due to a limited understanding of the viral life cycle and molecular mechanism of pathogenicity, there are currently no FDA-approved therapeutics or vaccines that exist against HFRS or HCPS, and only the whole virus-inactivated vaccine against HTNV or SEOV is available in China and Korea (Liu et al., 2019).

Increasing evidence shows that protein kinases play important roles in the pathogenesis of many illnesses, such as virus infection diseases. On one hand, the host kinases mediate multiple signaling pathways in response to the viral infection stimuli. On the other hand, every step of the replication cycle of viruses depends on host factors, such as PKC, ERK, PI3K, and FAK, which can regulate viral entry and replication (Wang et al., 2013; Meineke et al., 2019; Fedeli et al., 2020). Furthermore, the virus can hijack a large number of host kinases at distinct steps of their life cycles (Schor and Einav, 2018). However, only a few previous studies have reported on the function of protein kinases involved in HTNV infection. Thus, a better understanding of the molecular dialogs between HTNV and host protein kinases will help to improve the development of novel therapeutic approaches and highlight the role of protein kinase inhibitors in regulating HTNV infections.

To address this, we evaluate the *in vitro* anti-HTNV activity of a total of 901 kinase inhibitors in this study, observe the therapeutic potential of candidates to combat HTNV infection, and confirm the role of identified candidate kinase inhibitor. The feasibility of targeting kinase inhibitors is steadily moving from bench to clinic and already-approved drugs, and potentially be repurposed for treatments of HTNV infections.

## Materials and Methods

### Cells and Virus Propagation

A549 cells and Vero E6 cells were preserved in our laboratory and stored at −80°C, and were grown and maintained in Dulbecco's modified Eagle medium (DMEM) supplemented with 10% heat-inactivated FBS in a 5% CO_2_ incubator at 37°C. HTNV strain 76-118, preserved in our laboratory stock, was propagated in Vero E6 cells (a green monkey kidney epithelial cell line). For HTNV titer measurement, the median tissue culture infective dose (TCID50) of the virus was determined by immunofluorescence assay (IFA) through the Reed-Muench formula. For infection, A549 cells were rinsed with DMEM, and cells were infected at a multiplicity of infection (MOI) of 1. All related experiments with the HTNV were performed in biosafety level 2 (BSL-2) facilities, in accordance with the institutional biosafety operating procedures (Ma et al., 2017a).

### Kinase Inhibitor Treatment

Hantaan virus infection A549 cells were used to screen for anti-hantaviral kinase inhibitors. The kinase inhibitor library was purchased from Selleck. To screen and confirm the anti-HTNV kinase inhibitors, A549 cells were seeded in 96-well plates at a density of 1 × 10^4^ cells in a total volume of 100 μl per well and incubated overnight at 37°C and 5% CO_2_. Cells that reached 70–80% confluency were treated with the kinase inhibitors at a final concentration of 10 μM or DMSO (0.1%) with infected HTNV at an MOI of 1. After incubating for 4 h at 37°C, the supernatant was discarded, and the cells were cultured in DMEM supplemented with 2% FBS at 37°C in 5% CO_2_.

### Immunofluorescence Assay

After 96 h post-infection (hpi), HTNV-infected A549 cells were rinsed with PBS two times and fixed with ice-cold 4% paraformaldehyde (PFA) in PBS for 30 min at room temperature (RT) and permeabilized by the treatment of 0.1% Triton X-100 for 20 min at RT. Then, cells were treated with 3% bull serum albumin (BSA) at 37°C for 1 h and stained with mouse monoclonal antibodies 1A8 for the HTNV-NP (NP, 1:1,000, prepared by our laboratory) and incubated overnight. After three washes with PBS, the pre-stained cells were incubated with secondary Cy3-conjugated anti-mouse IgG (1:400) at 37°C for 1 h and cell nuclei were stained with DAPI (4′,6-diamidino-2-phenylindole, 1:5,000, Sigma). Samples were observed using an inverted fluorescence microscope (Olympus, Japan). The intensity was measured by an Infinite 200 PRO microplate reader (TECAN, Switzerland; Ma et al., 2017b).

### Inhibition Rate Measurement

The high-throughput screen (HTS) method has been established in our laboratory previously (Li et al., 2021). In brief, the signals of NP intensity and DAPI intensity were measured separately, and the infection rate was calculated by the following formula: NP signal intensity/DAPI signal intensity × 100%. Subsequently, the HTNV inhibition rate after kinase inhibitors treatment was obtained, inhibition rate (%) = [(Ac–As)/(Ac–Ab)] × 100, in which Ac referred to the infection rate of Vehicle controls, whereas As referred to HTNV-infected cells treated with the kinase inhibitors, and Ab referred to the blank control value of uninfected. Kinase inhibitors were selected with an inhibition rate of more than 80% for follow-up studies. The *Z*′ factor was calculated using the following equation and determined to be 0.54, indicating that the primary screening was robust (Zhang et al., 1999).


$$\begin{array}{l} Z\prime =1-\frac{\left(3\sigma_{c+}+3\sigma_{c-}\right)}{\left|\mu_{c+}-\mu_{c-}\right|} \end{array}$$

### Cell Viability Assays

Cell viability was determined by using the cell counting kit-8 (CCK-8) assay (YEASEN, China), which is widely used in cell proliferation and cytotoxicity based on WST-8, a rapid and highly sensitive detection reagent. In the presence of an electron coupling reagent, WST-8 can be reduced by dehydrogenase in mitochondria to generate a highly water-soluble orange-yellow formazan product (formazan). The shade of color is proportional to cell proliferation and inversely proportional to cytotoxicity. First, A549 cells were seeded in 96-well plates and incubated for 24 h, and cells were subject to different concentrations of kinase inhibitors (1, 2.5, 5, 10, 25, 50, and 100 μM) for 48 h. After the treatments, cells were added with 10 μl of CCK-8 reagents at 37°C for 2 h of incubation. Then, the absorbance was measured at an optical density of 450 nm to determine the cell viability with a microplate reader (BioTek, Germany). The CC_50_ values, which is the concentration that results in 50% cell viability, were calculated using GraphPad Prism by interpolation.

### Quantitative Real-Time PCR (RT-PCR)

A549 cells grown in 6-well plates were infected with HTNV. Total RNA was extracted from A549 cells by using the RNA extraction kit (Axygen, USA) according to the manufacturer's procedures. For the analysis of HTNV-S expression, a Prime Script RT Master Mix (YEASEN, China) was used to transcribe RNA into cDNA. The quantity of RNA was performed by using a QuantiTect SYBR Green RT-PCR kit (YEASEN, China), cDNA was denatured at 95°C for 30 s and amplified for 45 cycles of 10 s at 95°C, 31 s at 60°C in the LightCycler 96 (Roche, Switzerland). The relative expression levels of HTNV-S mRNA were calculated using the comparative Ct (2^−ΔΔ^Ct) method. The sequence of a primer used in quantitative real-time PCR (qRT-PCR) was as follows: HTNV-S F-GAGCCTGGAGACCATCTG, R-CGGGACGACAAAGGATGT; β-actin F-5′-TGACGGGGTCACCCACACTG-3′, R-5′-AAGCTGTAGCCGCGCTCGGT-3′). The relative mRNA expression of HTNV-S was normalized to the expression of β-actin.

### Western Blot Analysis

After the kinase inhibitors treatments, protein from A549 cells was extracted by using the RIPA Lysis Buffer System containing protease and phosphatase inhibitors. The protein concentration was quantified and 30 μg of total proteins were subjected to gel electrophoresis and transferred to the polyvinylidene difluoride membrane (Millipore, Germany). After being blocked with 5% skimmed milk in TBS at room temperature (RT) for 2 h, the membrane was incubated with the primary antibodies at 4°C overnight. The primary antibody against HTNV-NP was diluted at 1:1,000, and the antibody for GAPDH (Abcam, UK) was diluted at 1:1,000. After extensive wash, the membranes were incubated with the secondary antibodies labeled with infrared dye at RT for 1 h. Finally, the protein bands were visualized by using the Odyssey Infrared Imaging System (Biosciences, USA).

### Statistics

All data were analyzed by GraphPad Prism Version 6.0 software. Data were expressed as the mean ± standard errors (SEs). A one-way analysis of variance (ANOVA) and *t*-test were used for statistical evaluations. All the experiments were repeated at least three times for each group. A *p*-value of < 0.05 was considered to be statistically significant. The dose–response curve was created by the nonlinear regression model. Both EC_50_ and CC_50_ were calculated using GraphPad Prism 6.0 software.

## Results

### Screening of the Possible Anti-HTNV Protein Kinase Inhibitors Without Significant Cytotoxicity

**Figure 1:**
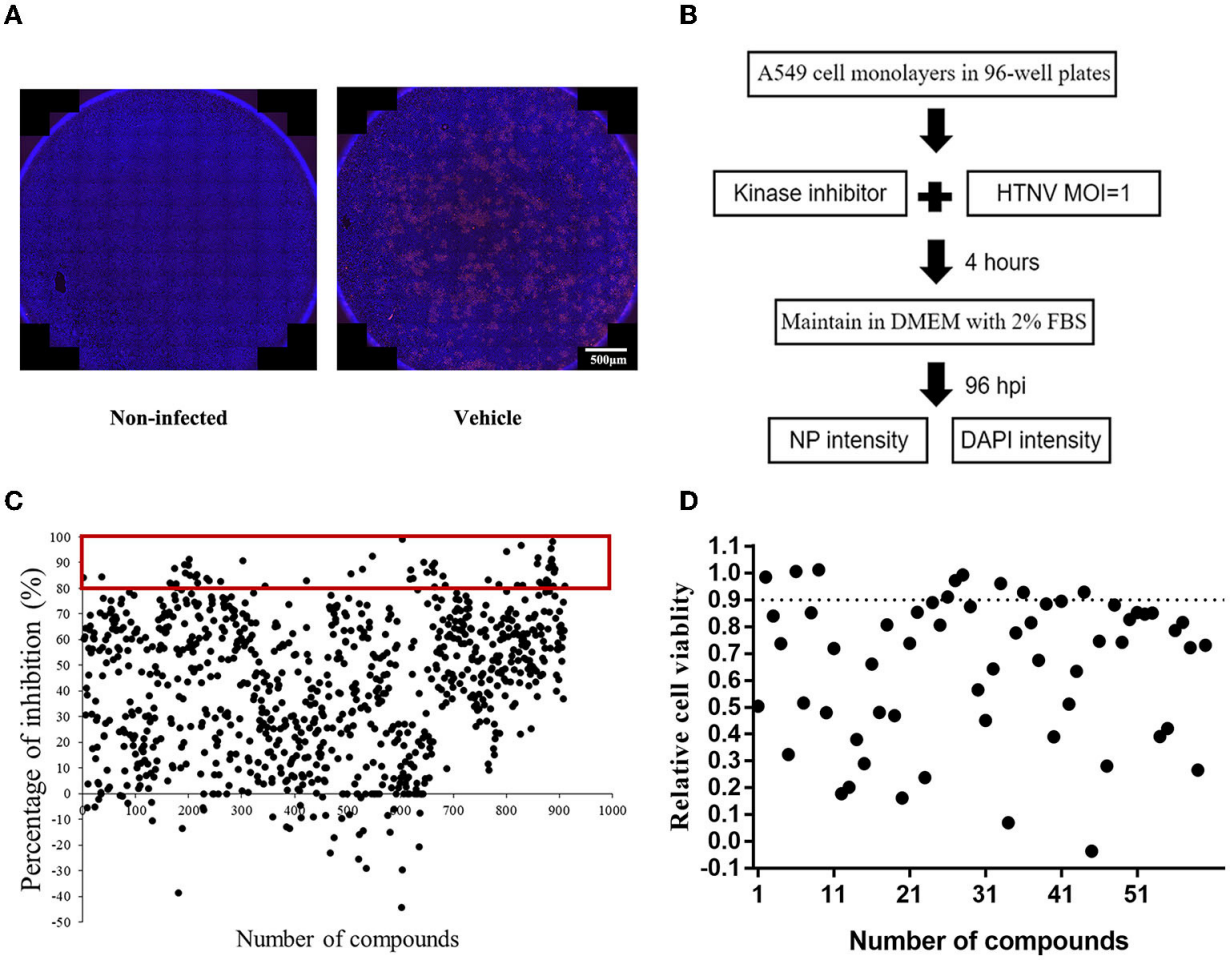
The screening of protein kinase inhibitors with anti-Hantaan virus (anti-HTNV) activity. **(A)** Observation of the anti-HTNV activity of kinase inhibitors by immunofluorescence assay (IFA), red staining for the expression of nucleocapsid protein (NP), and blue staining for the cell nuclei, scale bar = 500 μm. **(B)** Preliminary high-throughput screening of protein kinase inhibitors with anti-HTNV activity. **(C)** Among 901 protein kinase inhibitors, 60 could inhibit the HTNV more than 80%. **(D)** Among the above 60 protein kinase inhibitors, 10 compounds had no significant effect on cell viability.

To identify the potential small-molecule compounds with anti-HTNV activity, we screened the bioactivity of 901 kinase inhibitors against HTNV by an immunofluorescence high-throughput screen (HTS) assay, and the expression of virus nucleocapsid protein (NP) was stained with red immunofluorescence (Figures 1A,B). The screening result showed that the inhibitory percentage of 60 compounds against HTNV was more than 80% (Figure 1C). Furthermore, considering that safety is very necessary for the potential candidates, we evaluated the effect of these compounds on the cell viability and found that 10 of them in a concentration of 10 μM had no significant effect on the A549 cell viability (Figure 1D).

### Identification of Potential Candidates of Kinase Inhibitors With *in vitro* Anti-HTNV Activity

**Figure 2:**
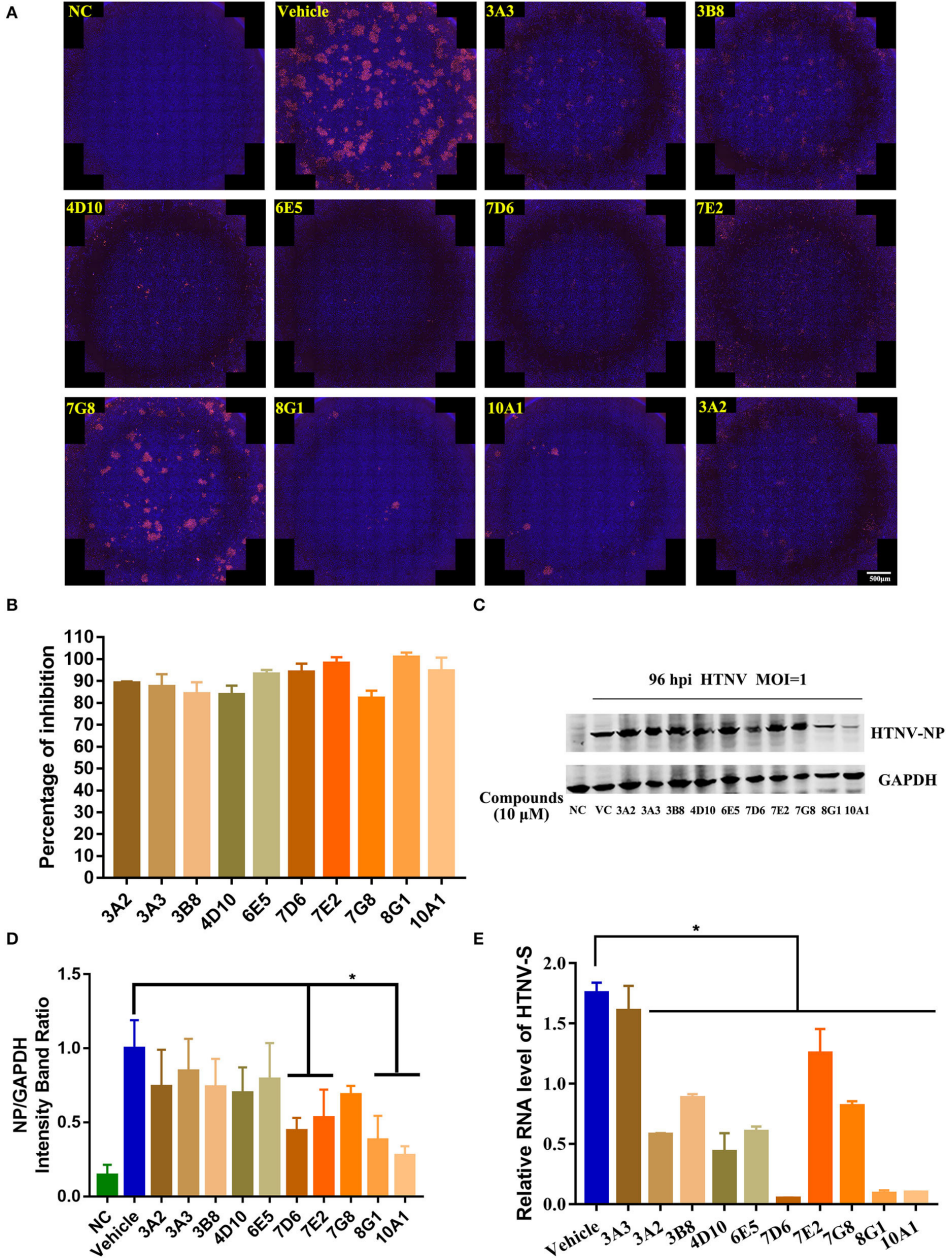
Identification of potential kinase inhibitor candidates with *in vitro* anti-HTNV activity. **(A)** Immunofluorescence staining of HTNV nucleocapsid protein after the 10 protein kinase inhibitors treatment, NC, negative control, Vehicle: Vehicle-treated control, scale bar = 500 μm. **(B)** Inhibitory percentage of protein kinase inhibitors to virus infection based on the immunofluorescence staining. **(C)** The representative Western blot staining for HTNV NP protein in the negative control (NC) group, Vehicle-treated control (VC) group, and 10 μM 10 protein kinase inhibitors treatment groups. **(D)** The analyzed result of expression of HTNV nucleocapsid protein in the NC, VC, and 10 protein kinase inhibitors treatment groups in A549 cells post HTNV infection, *p < 0.05 vs. Vehicle, *n* = 3. **(E)** Inhibitory activity of protein kinase inhibitors to the expression of HTNV-S gene extracted from intracellular RNA, the mRNA expression level of HTNV-S gene was normalized to the respective β-actin and analyzed, *p < 0.05 vs. Vehicle, *n* = 3.

Next, to confirm the *in vitro* antiviral activity against the HTNV of these 10 candidates, the inhibitory effect of these compounds on HTNV nucleocapsid protein and HTNV-S gene expression was observed. The NC group refers to A549 cells treated with 0.1% DMSO without HTNV infection during the experiment, while the Vehicle group refers to the cells infected by HTNV with 0.1% DMSO treatment. Inconsistent with the screening result, all the tested compounds had significant inhibition of the HTNV by IFA assay (Figures 2A,B). The inhibitory effect of the 10 protein kinase inhibitors on the nucleocapsid protein was measured by the Western blot staining, and the results confirmed that 10 μM compound 7D6, compound 7E2, compound 8G1, and compound 10A1 had the most significant inhibitory effect against the HTNV nucleocapsid protein (Figures 2C,D). Interestingly, qRT-PCR results showed that compound 3A3 was not able to inhibit the expression of the HTNV-S gene, but three kinase inhibitors, such as compound 7D6, compound 8G1, and compound 10A1 could significantly inhibit the expression of HTNV-S genes (Figure 2E). These data indicated that compound 7D6, compound 8G1, and compound 10A1 had potent *in vitro* anti-HTNV activity, and simultaneous inhibition of NP and HTNV-S gene synthesis, and these compounds are the potential candidates for kinase inhibitors against HTNV infection.

### Compound 8G1 With More Potent Inhibitory Activity Against HTNV and Less Toxicity

**Figure 3:**
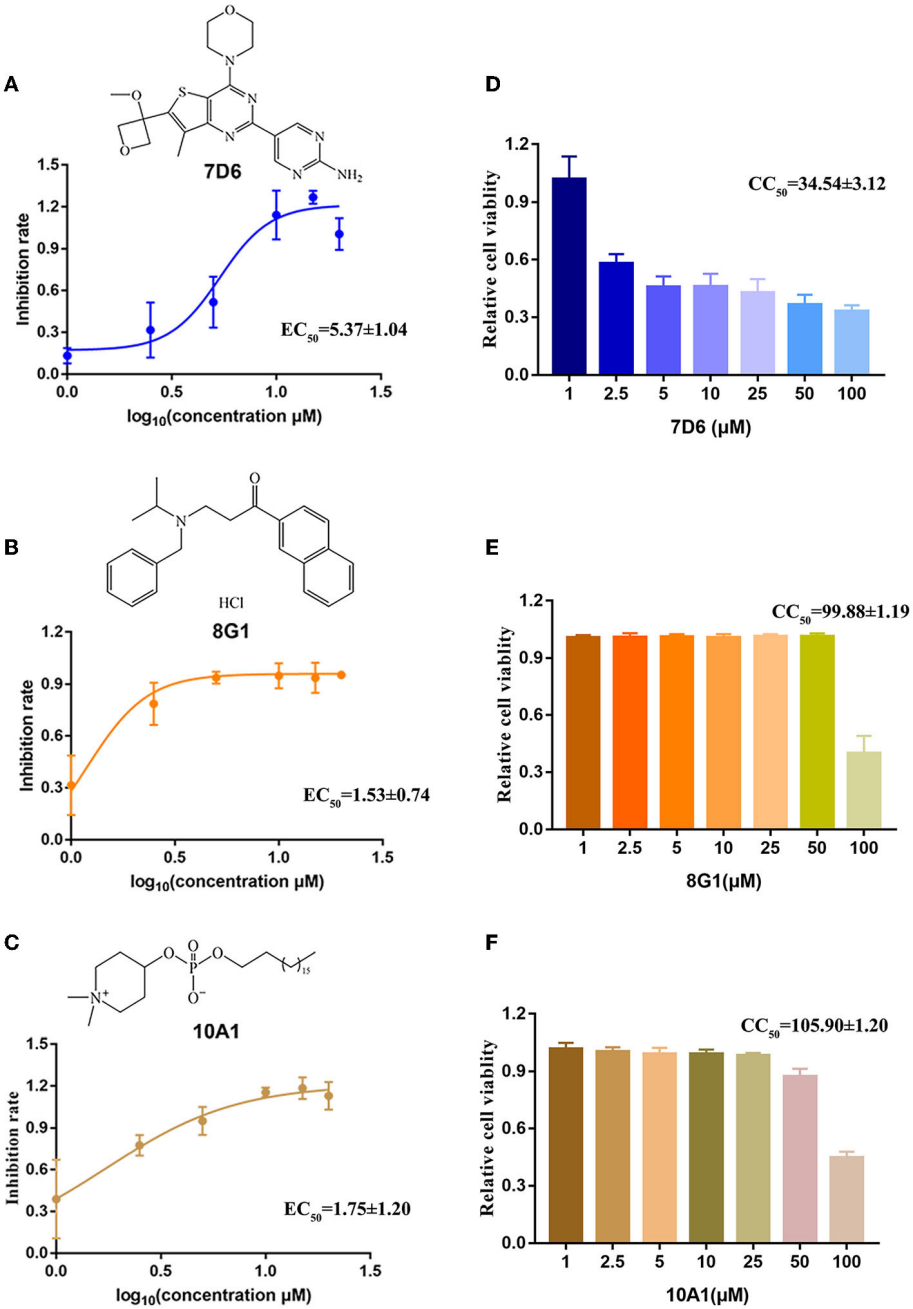
Evaluation of the selectivity index of three kinase inhibitor candidates. The chemical structure and dose-dependent inhibitory activity of **(A)** compound 7D6, **(B)** compound 8G1, and **(C)** compound 10A1 against HTNV at concentrations ranging from 1 to 20 μM. The half cytotoxicity concentration of **(D)** compound 7D6, **(E)** compound 8G1, and **(F)** compound 10A1 was measured at a concentration ranging from 1 to 100 μM, *n* = 3.

To further confirm the inhibitory activity of the three protein kinase inhibitor candidates against HTNV, we then measured the inhibitory effect of compound 7D6, compound 8G1, and compound 10A1 at concentrations ranging from 1 to 20 μM against HTNV (MOI = 1) *in vitro*. The results showed that all three compounds displayed an anti-HTNV activity in a dose-dependent manner, and the concentration for 50% of maximal effect (EC_50_) of compound 7D6, compound 8G1, and compound 10A1 was 5.37 ± 1.04, 1.53 ± 0.74, and 1.75±1.20 μM, respectively (Figures 3A–C). Both efficacy and safety are required for the candidate compound, and the selectivity index (SI) is a ratio that measures the window between cytotoxicity and antiviral activity. A high SI indicates that a drug will be safe and effective in the clinic. Thus, we measured the half cytotoxicity concentration (CC_50_) of three candidate compounds on the A549 cells by CCK-8 staining. The results showed that the CC_50_ of compound 7D6, compound 8G1, and compound 10A1 was 34.54 ± 3.12, 99.88 ± 1.19, and 105.90 ± 1.20 μM, respectively (Figures 3D–F). Based on the values of EC_50_ and CC_50_, the SI of compound 7D6, compound 8G1, and compound 10A1 were 6.43, 65.28, and 60.51, respectively. These results indicated that compound 8G1 had the highest SI among the three candidates.

### Inhibitory Activity of Compound 8G1 to HTNV Replication in A549 Cell Post-Infection

**Figure 4:**
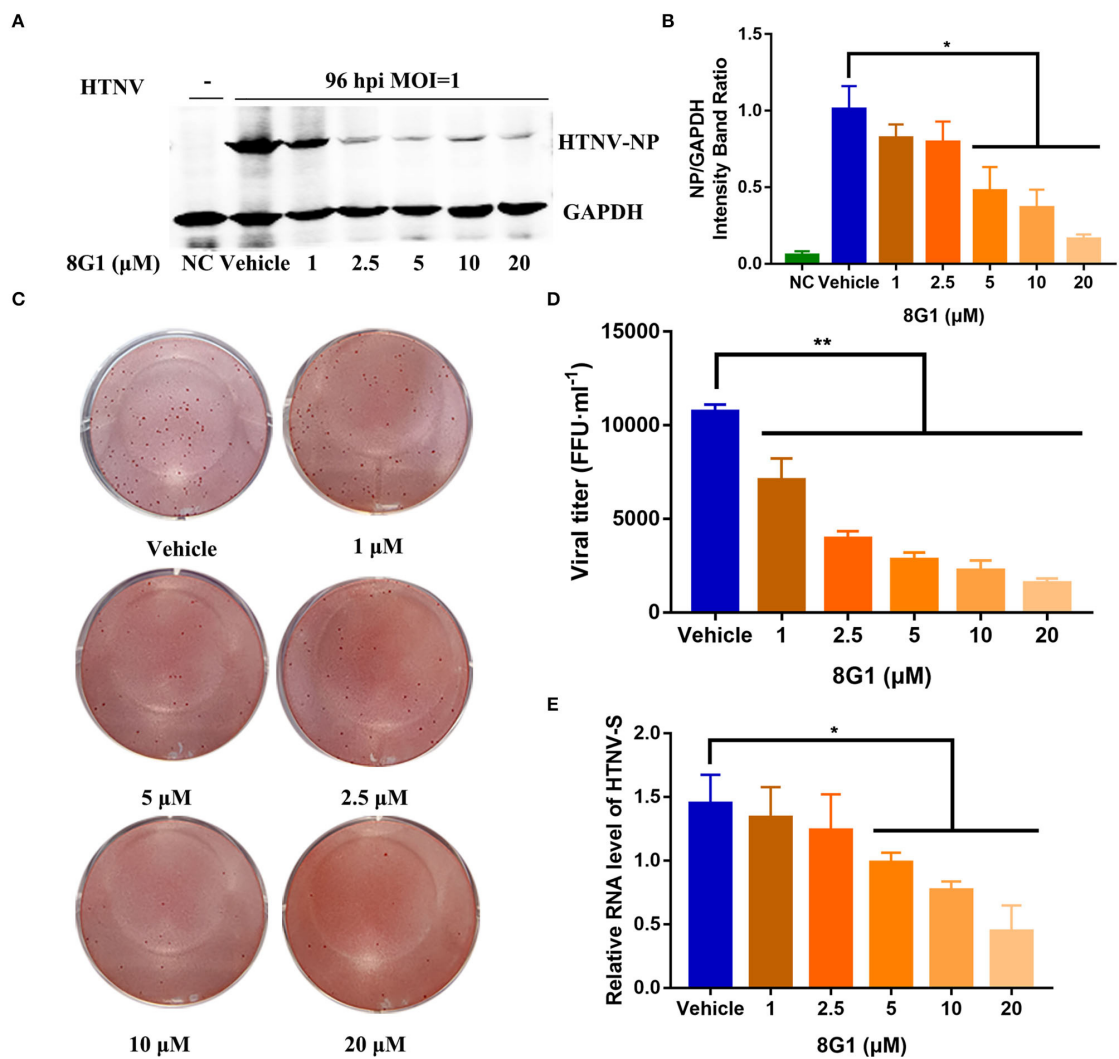
Compound 8G1 inhibited HTNV replication in A549 cell post-infection. **(A)** The representative Western blot staining for HTNV NP protein in the negative control (NC) group, Vehicle-treated control group, and compound 8G1 (1, 2.5, 5, 10, and 20 μM) treatment groups. **(B)** The analyzed result of expression of HTNV nucleocapsid protein in the NC, Vehicle, and compound 8G1 treatment groups in A549 cells post-HTNV infection, *p* < 0.05 vs. Vehicle, *n* = 3. **(C)** The representative imaging for the focus-forming unit of HTNV in the Vehicle group and compound 8G1 (1, 2.5, 5, 10, and 20 μM) treatment groups. **(D)** The viral titer of HTNV was analyzed in the Vehicle group and compound 8G1 treatment groups. **p < 0.01 vs. Vehicle, *n* = 3. **(E)** The expression of HTNV-S gene in the Vehicle group and compound 8G1 (1, 2.5, 5, 10, and 20 μM) groups, *p < 0.05 vs. Vehicle, *n* = 3.

After identification of compound 8G1 as a potent anti-HTNV protein kinase inhibitor with less cytotoxicity, we then investigated whether this compound could inhibit the replication of HTNV in infected A549 cells. After HTNV infection, A549 cells were cultured in the medium with compound 8G1 at a concentration of 10 μM. The expression of HTNV protein and viral titer in HTNV-infected A549 cells were measured after compound 8G1 treatment at 96 hpi, the medium was not changed over the 96 hpi. Compared with the untreated control cells, the Western blot (Figures 4A,B) assay showed that compound 8G1 ranging from 1 to 20 μM could inhibit the expression of HTNV-NP in a dose-dependent model at 96 hpi. Meanwhile, the viral titer of HTNV was also significantly reduced after compound 8G1 treatment in a dose-dependent manner (Figures 4C,D), whereas only 5, 10, and 20 μM compound 8G1 treatments could reduce the expression of the HTNV-S gene significantly (Figure 4E). Exploration of the Therapeutic Regimen of Compound 8G1 Against HTNV Infection.

**Figure 5:**
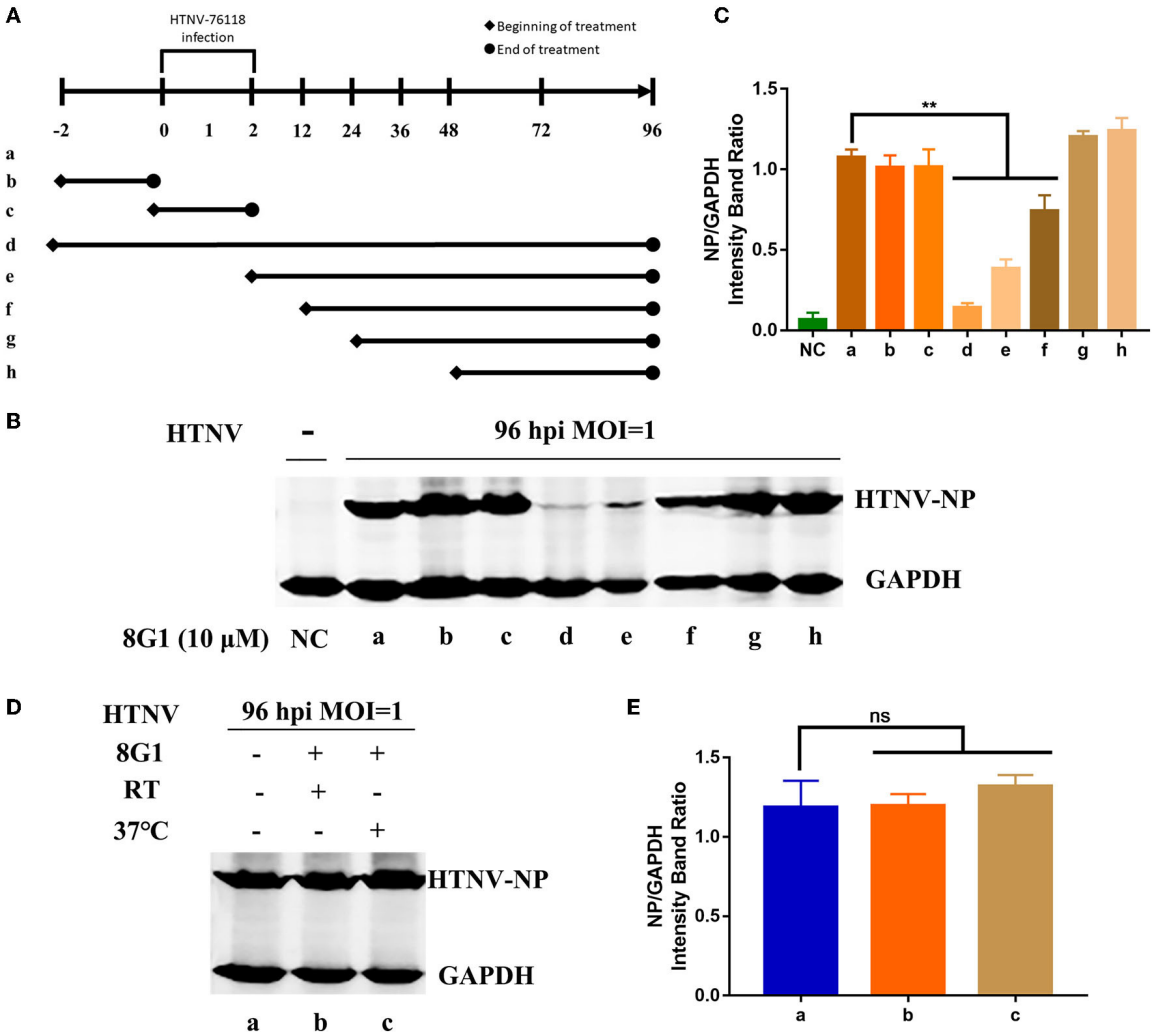
Compound 8G1 reduced the expression of HTNV-NP proteins at the early stage of HTNV infection. **(A)** Different administration regimens of compound 8G1 treated the HTNV-infected A549 cells at an MOI of 1.0. Lane a: without compound 8G1 treatment; lane b: compound 8G1 treatment for 2 h pre-infection; lane c: compound 8G1 treatment for 2 h post-infection (hpi); lane d–h: treatment continues from 2 h pre-infection, 2, 12, 24, and 48 hpi to the 96 hpi time point. **(B)** The representative Western blot staining for HTNV NP protein in the groups with different administration regimens of compound 8G1. **(C)** The analyzed result of the expression of HTNV-NP protein in groups with different administration regimens of compound 8G1. **p < 0.01 vs. a, *n* = 3. **(D)** The representative Western blot staining for HTNV NP protein in the A549 cells without or in the presence of compound 8G1 at different temperatures. **(E)** The analyzed result of the expression of HTNV NP protein in control or compound 8G1 treatment groups at room temperature (RT) or 37°C.

To explore the possible therapeutic regimen of compound 8G1 against HTNV infection, we then designed seven different administration regimens of compound 8G1 in HTNV-infected A549 cells. These are illustrated in Figure 5A. Mainly: (1) before HTNV infection, A549 cells were incubated with compound 8G1 for 2 h, which was then washed out; (2) during HTNV infection, compound 8G1 was added into A549 cells, the supernatant of which was removed after infection; (3) compound 8G1 was added at 2, 12, 24, and 48 hpi after HTNV-infected A549 cells, and the HTNV inhibition was observed. Compared with the infected cells (a), the expression of HTNV-NP was only reduced efficiently in three administration regimens of compound 8G1 in HTNV-infected A549 cells, namely, the regimen of 2 h pre-infection to 96 hpi, from 2 to 96 hpi, and from 12 to 96 hpi (Figures 5B,C). These results suggest that the administration of compound 8G1 at the early stage of HTNV infection could inhibit the replication and production of infectious HTNV in host cells. Next, we treated the HTNV with 10 μM compound 8G1 for 2 h at RT or 37°C to confirm whether the compound 8G1 had a direct virucidal effect on the HTNV. During the experiment, we selected three equal amounts of HTNV (MOI = 1), lane a was control A549 cells without any treatment, lane b and lane c were the HTNV treated by 10 μM compound 8G1 and incubated at room temperature or 37°C for 2 h, then the cells were infected by HTNV, and replaced with normal medium after infection, the samples were collected at 96 hpi for detection. Contrary to the control (a), the Western blot assay showed that compound 8G1 treatment could not affect the expression of HTNV NP at 96 hpi (Figures 5D,E). This result indicated that compound 8G1 inhibited the HTNV through indirect virucidal activity.

### Molecular Recognition of AKT by Compound 8G1

**Figure 6:**
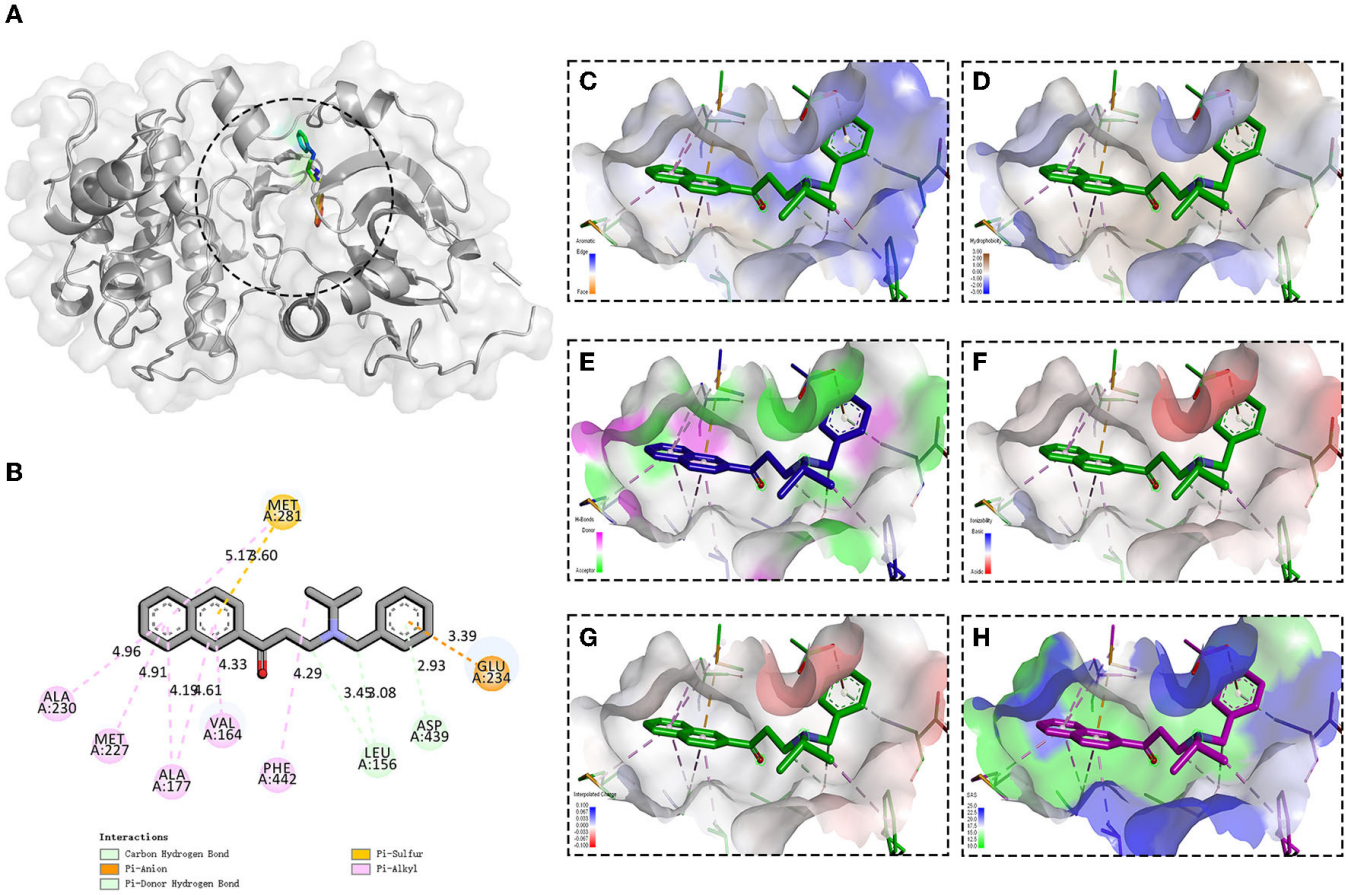
Binding site and model of compound 8G1 with AKT. **(A)** Overview of the docked pose between the compound 8G1 and binding pocket to the active site of Akt. **(B)** The close view of the interaction between compound 8G1 and the active site of Akt. **(C)** The analyzed result of the aromatic ring edges or faces. **(D)** The analyzed result of the hydrophobicity. **(E)** The analyzed result of the hydrogen bond. **(F)** The analyzed result of the ionizability. **(G)** The analyzed result of the atomic charge, values less than −0.1 are mapped in red, and values larger than +0.1 are mapped in blue. **(H)** The analyzed result of the solvent accessibility surface, small values (green) correspond to buried residues, whereas large values (blue) correspond to exposed residues.

It has been reported that HTNV infection could cause the activation of serine/threonine-protein kinase B (PKB, also known as AKT, PDB ID: 4EKL), especially AKT ser473 (Yu et al., 2014), so we then investigated whether the compound 8G1 could target the AKT signaling during HTNV infection. The molecular docking results showed that compound 8G1 formed interactions with the key amino acid residues at its active site, and revealed several molecular interactions responsible for the observed affinity: (i) carbon-hydrogen bond and pi-donor hydrogen bond interactions between the carbon atom and benzene ring with LEU156 and ASP439; (ii) pi-anion interactions between the benzene ring and GLU234; (iii) pi-sulfur interactions between the naphthalene ring and MET281; and (iv) pi-alkyl interaction between the naphthalene ring and carbon atom with ALA230, MET227, ALA177, VAL164, MET281, and PHE442. In addition, the binding surface model of compound 8G1 and Akt1 was analyzed, such as the aromatic ring edges or faces, hydrophobicity, hydrogen bond, ionizability, atomic charge, and solvent accessibility surface (Figure 6). Involvement of AKT/Mammalian Target of Rapamycin (MTOR) Signaling Pathway in the Anti-HTNV Mechanism of Compound 8G1.

**Figure 7:**
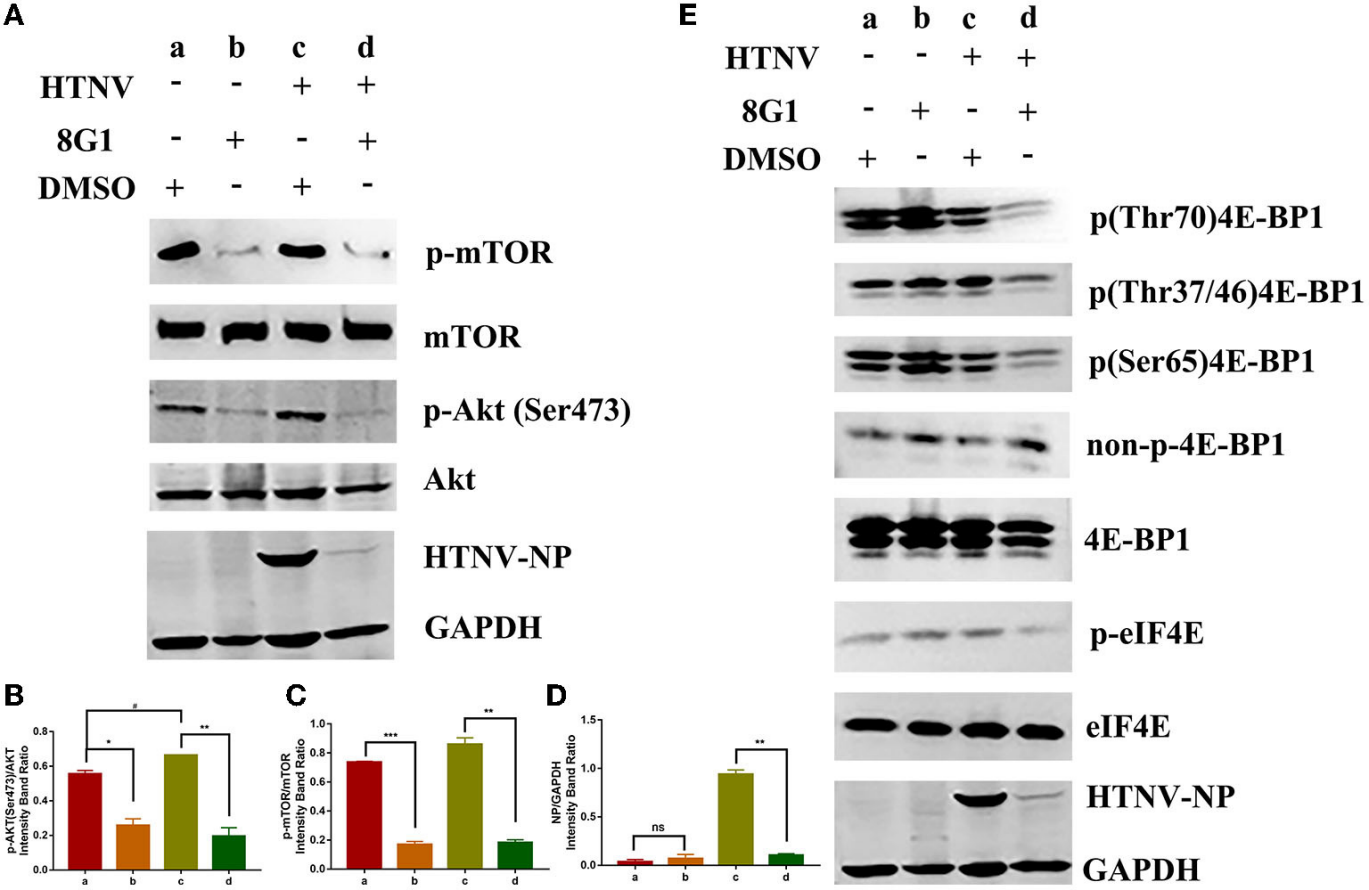
Effect of compound 8G1 to the protein kinase B (Akt)/mammalian target of rapamycin (mTOR) pathway. **(A)** The representative Western blot staining for phosphorylation of Akt and mTOR proteins in A549 cells. **(B)** The analyzed result of the expression of p(Ser473) Akt, *p < 0.05, ^#^*p* < 0.05 vs. DMSO treated group, **p < 0.01 vs. HTNV group, *n* = 3. **(C)** The analyzed result of the expression of p-mTOR, **p < 0.01 vs. DMSO treated group, ***p < 0.005 vs. HTNV group, *n* = 3. **(D)** The analyzed result of the expression of HTNV-NP, **p < 0.001 vs. HTNV group, *n* = 3. **(E)** A549 cells were with treated 10 μM 8G1 or DMSO infected in the presence of inhibitors for 96 h before cells were harvested. The phosphorylation status of 4E-BP1 and elF4E was analyzed using antibodies against total 4E-BP1, p-4E-BP1 (Thr70), p-4E-BP1 (Ser65), p-4E-BP1 (Thr37/46), non-p-4E-BP1 (Thr46), p-eIF4E, and eIF4E. Total protein was isolated and analyzed by the Western blot using antibodies as shown. ^a^A549 cells treated with solvent DMSO in the absence of HTNV infection; ^b^A549 cells treated with compound 8G1 in the absence of HTNV infection; ^c^A549 cells treated with the solvent DMSO in the context of HTNV infection; ^d^A549 cells treated with compound 8G1 in the context of HTNV infection.

Since the compound 8G1 could bind to the protein kinase Akt, we hypothesized that the phosphorylated conformation of Akt would be hampered. We then observed the effect of compound 8G1 on the expression of phosphorylated Akt in A549 cells. Compared with the Vehicle (DMSO) treatment, the expression of p(Ser473)Akt and HTNV-NP proteins was augmented in HTNV-infected cells, and compound 8G1 could inhibit the expression of p(Ser473)Akt and HTNV-NP proteins significantly (Figures 7A,B,D). Because the mammalian target of rapamycin (mTOR) is the main molecule downstream of the Akt signaling pathway, we then evaluated the inhibition of phosphorylation by compound 8G1 in HTNV infection. Consistently, compound 8G1 also reduced the p-mTOR level during HTNV infection (Figures 7A,C).

It was reported that the Akt/mTOR pathway was required for eukaryotic translation initiation factor 4E (eIF4E) assembly and viral protein synthesis during virus infection (Hayashi et al., 1981). Thus the effect of compound 8G1 on the expression of eIF4E phosphorylation and HTNV NP synthesis was examined after HTNV infection. The results showed that phosphorylation of eIF4E, as well as HTNV NP synthesis, was reduced upon the compound 8G1 treatment and HTNV infected A549 cells at 96 hpi. Meanwhile, eIF4E-binding protein 1 (4E-BP1) is a well-known substrate of the mechanistic target of mTOR, and the phosphorylation of 4E-BP1 causes its release from eIF4E to allow cap-dependent translation to proceed. A decreased phosphorylation of both (Thr37/46) 4E-BP1 and (Ser65) 4E-BP1 levels was observed after compound 8G1 treatment in HTNV-infected cells at 96 hpi (Figure 7E). Collectively, these results suggest that compound 8G1 could inhibit the Akt/mTOR signaling pathway, lead to a decreased level of eIF4E and 4E-BP1 phosphorylation, and therefore impact the viral nucleocapsid protein synthesis in HTNV-infected cells.

## Discussion

Emerging viral infections represent a major global concern for public health, and ongoing virus replication and prolonged drug exposure lead to the selection of resistant strains (Rumlova and Ruml, 2018), so there is an urgent need for the development of new antiviral drugs. At present, ~118 antiviral drugs have been approved by the FDA for the treatment of diseases caused by various viruses except for the HTNV, and among the FDA-approved antiviral agents, more than 90% of current on-the-market drugs are comprised of small molecules (Tompa et al., 2021). The future important role of small molecules to build a new portfolio of drugs to solve the viral infection challenges is focused widely.

Since dysregulation and mutations of protein kinases play causal roles in human disease including the infection disease, this family of enzymes has become one of the most important drug targets over the past two decades (Roskoski, 2016), and the spectacular results demonstrated with imatinib mesylate lead to the development of a flurry kinase inhibitors offering clinical benefit (Koliopoulos, 1984; Protein Kinase Inhibitors, 2012). In this study, 60 of the total tested 901 protein kinase inhibitors present remarkable anti-HTNV activity. This result sheds light on the development of small molecule drugs for the treatment of HTNV infection.

In drug preclinical development, the right balance between pharmacological activity and safety should be considered for the lead candidate discovery. The selectivity index is the ratio of the toxic concentration of a lead compound against its effective bioactive concentration, and its value ≥ 10 was assumed to belong to a selected potential sample that can be further investigated (Indrayanto et al., 2021). We determined that the SI value of compound 8G1 was 65.28, it was much higher than the coumarin compounds against HTNV investigated previously (Li et al., 2021), or the FDA-approved kinase inhibitor sorafenib, which reduced the replication of Venezuelan equine encephalitis virus and other alphaviruses (Lundberg et al., 2018). The results indicated that compound 8G1 has considerable potential as a novel agent against HTNV.

To elucidate the effects of compound 8G1 in the course of HTNV infection, we used molecular docking to predict the interaction between compound 8G1 and protein kinase, suggesting that coumpound 8G1 inhibited HTNV replication by suppressing the host protein kinase. We first demonstrated that compound 8G1 inhibits the phosphorylation of AKT, and the expression of phosphorylated mTOR and downstream molecules was also reduced after compound 8G1 treatment. Considering that the activation of AKT leads to the phosphorylation and activation of molecules, benefits viral mRNA translation *via* interaction of the viral N protein and host eIF4E (Zhan et al., 2020). These results suggest that compound 8G1 inhibits complex eIF4F formation and synthesis of N protein by inhibiting the AKT/mTOR signaling pathway. Interestingly, inhibiting the expression level of N protein can further slowdown the viral mRNA replication process, thereby enhancing the anti-HTNV effect of compound 8G1 (Vera-Otarola et al., 2021). Interestingly, we found that there is no obvious reduction in phosphorylated 4E-BP1 after compound 8G1 treatment in the absence of HTNV infection, while a significant decrease was observed in phosphorylated mTOR. Although this result was not consistent with the mechanism that 4E-BP1 was a direct target of mTOR, in some cases, it was also reported that 4E-BP1 was phosphorylated in an mTOR-independent manner in some cancer cells (Zhang and Zheng, 2012). It indicated that a similar situation might happen in A549 cells, which was a human alveolar adenocarcinoma cell line. However, this speculation needs future confirmation. These results may help to design novel targets for therapeutic intervention against HTNV infection.

In conclusion, our current study identified a protein kinase inhibitor with potent anti-HTNV activity and low cytotoxicity and provides insight into the possible molecular mechanism of this candidate. However, further effort is warranted to elucidate the relationship between the chemical structure of kinase inhibitors and biological activity against the HTNV. This will help to provide new targets and lead compounds for safe and effective antiviral drug discovery.

## Data Availability Statement

The original contributions presented in the study are included in the article/Supplementary Material, further inquiries can be directed to the corresponding authors.

## Author Contributions

ZL designed the study and conducted the experiments drafting the manuscript. FW and QY performed data analysis. DK wrote the manuscript. XZ and YD performed the immunofluorescence assay. YL, DZ, ZC, MJ, and XX discussed the results and performed data analysis. ML and XW guarantor of integrity of the entire study, manuscript revision, and final approval for submission. All authors reviewed the results, commented on the manuscript, and approved the final version of the manuscript.

## Funding

This project was supported by the Foundation for the Scientific and Technological Project in Shaanxi Province (Nos. 2019ZDLSF02-03 and 2021JM-219) and the funding of Air Force Military Medical University (No. 2018JSTS08).

## Conflict of Interest

The authors declare that the research was conducted in the absence of any commercial or financial relationships that could be construed as a potential conflict of interest.

## Publisher's Note

All claims expressed in this article are solely those of the authors and do not necessarily represent those of their affiliated organizations, or those of the publisher, the editors and the reviewers. Any product that may be evaluated in this article, or claim that may be made by its manufacturer, is not guaranteed or endorsed by the publisher.

## Abbreviations

HTNV: Hantaan virus
MOI: multiplicity of infection
IFA: Immunofluorescence assay
AKT: protein kinase B
HTS: high-throughput screen
NP: nucleocapsid protein
SI: selectivity index.
